# Extract from* Bougainvillea xbuttiana* (Variety Orange) Inhibits Production of LPS-Induced Inflammatory Mediators in Macrophages and Exerts a Protective Effect* In Vivo*

**DOI:** 10.1155/2019/2034247

**Published:** 2019-03-05

**Authors:** Rodolfo Abarca-Vargas, Vera L. Petricevich

**Affiliations:** Facultad de Medicina, Universidad Autónoma del Estado de Morelos, Calle Leñeros Esquina Iztaccíhuatl s/n. Col. Volcanes, 62350 Cuernavaca, MOR, Mexico

## Abstract

**Background:**

Different pharmacological properties, such as antioxidant, antiproliferative, and anti-inflammatory properties, have been described among natural products. We previously described that the* Bougainvillea xbuttiana* (Variety Orange) ethanolic extract (BxbO) has an anti-inflammatory effect; however, this action is not fully understood. In this study, the action of the BxbO extract on the secretion of inflammatory mediators in two experimental models,* in vitro* and* in vivo*, after LPS challenge was evaluated.

**Methods:**

Peritoneal macrophages were obtained from female BALB/c mice and LPS-challenged with or without the BxbO extract. For the evaluation of mediators, the supernatants at 0, 12, 24, 36, and 48 hours were collected. For* in vivo* estimation, groups of female BALB/c mice were first intraperitoneously injected with different amounts of LPS and later administered the oral BxbO extract (v.o.) for 144 hours. To understand the mechanism of action, sera obtained from mice were collected at 0, 2, 4, 8, 12, and 24 hours after LPS challenge (with or without BxbO) for the detection of mediators.

**Results:**

The results showed that, in both peritoneal macrophages and sera of mice treated with the BxbO extract 1 hour before or together with LPS challenge, proinflammatory cytokines and nitric oxide release were unquestionably repressed. In contrast, in both systems studied here, the IL-10 levels were elevated to 5 to 9 times. At lethal doses of LPS, the BxbO extract treatment was found to protect animals from death.

**Conclusions:**

The results revealed that the inhibitory, protective, and benign effects of the BxbO extract were due to its capacity to balance the secretion of mediators.

## 1. Background

Currently, several studies have demonstrated the immunosuppressive activities of different natural products. This activity of natural products results in important applications for various disease therapies. Some products derived from plants are able to diminish the production of inflammatory mediators, suggesting their value for the treatment of autoimmune diseases. To better understand cytokine production, different interventions are performed to be involved with the host response. At the same time, the results suggest specific antigen blockade, and it has been speculated that inhibiting endogenous cytokines in distinct preparations could be useful as part of therapeutic strategies for different diseases [1]. The human immune system plays a decisive role in the protection of the body against pathogenic agents. The function of T cells is to recognize antigens on the surface of antigen-presenting cells and secrete cytokines. The presence of cytokines in the medium will determine T helper (TH) differentiation. The TH response involves the activation of macrophages and cell-mediated immunity and is still capable of affecting the differentiation of immunoglobulins and secretion of antibodies [2]. TH cells differentiate into TH1 cells, which secrete proinflammatory cytokines, such as tumour necrosis factor (TNF-*α*), interleukin-2 (IL-2), and interferon-gamma (IFN-*γ*), whereas TH2 cells secrete the anti-inflammatory cytokines IL-4, IL-5, and IL-10 [2].

There are numerous anti-inflammatory drugs, including traditional medicines that have been studied to cure diseases. The action of the molecules on T cells can be estimated by tests performed* in vitro* when exposed to an antigen or mitogen. These molecules can stimulate T cells to start a cascade of events such as proliferation and differentiation. The process of proliferation leads to the activation of certain transcription factors and the synthesis and secretion of cytokines [3]. Numerous studies have shown the effect of different molecules on the blockade of cytokine synthesis. However, to date, there are no specific therapies available.

The inflammatory process is correlated with an increase in proinflammatory cytokine activity [4]. Treatment of distinct hosts with the lipopolysaccharide endotoxin (LPS) causes inflammation, which is correlated with excessive inflammatory mediator secretion. Sepsis is a frequent clinical problem that involves a systemic inflammatory response that causes disproportionate secretion, which damages the cell, followed by multiorgan failure and death [5–7]. Septic shock is correlated with proinflammatory cytokines, like interleukin-1 (IL-1*β*), tumour necrosis factor (TNF-*α*), and nitric oxide (NO), as well as anti-inflammatory cytokines, such as IL-10 [8]. The most promising strategy for the treatment of endotoxic sepsis would be to suppress an excessive systemic inflammatory response. Thus, the use of agents able to regulate the release of inflammatory cytokines and increase the survival of endotoxaemia hosts would have potentially widespread therapeutic uses.

Currently, the treatment of sepsis in humans remains a major clinical challenge, and the most effective therapy is through the use of glucocorticosteroids. This therapy is used to suppress the functions of activated macrophages by reducing cytokine secretion, including IL-1*β* and TNF-*α*. Despite this therapy having effective actions, its adverse effects limit its clinical use [9]. For this reason, it is essential to search for anti-inflammatory agents with fewer side effects for better treatments. Several compounds obtained from plant products are being investigated, some of which have pharmacological activities specific to the treatment of inflammatory diseases, such as sepsis [9].

The central focus of this study was the* Bougainvillea* plant, which has recently received increased attention for its effects on the immune system [10–12]. In traditional Mexican medicine,* Bougainvillea* is used as a treatment in different types of diseases such as fever, headache, diarrhoea, and cough, among others [8]. In addition, we have previously described that the BxbO extract has shown different biological properties, among others the antioxidant, analgesic, anti-inflammatory and immunomodulatory activities [10]. Although there is substantial evidence of the biological activities of* Bougainvillea*, the mechanism of action is still not fully understood. The presence of different components was observed in the preparation of different coloured extracts of* Bougainvillea xbuttiana*. In this study, the action of the* Bougainvillea xbuttiana* (Variety Orange) (BxbO) ethanolic extract on sepsis was evaluated.

## 2. Materials

Actinomycin D, ethanol, phosphoric acid, 2,2′-azino-bis(3-ethylbenzothiazoline-6-sulfonic acid) (ABTS), naphthylenediamine (NADPH), dimethylformamide, lipopolysaccharide LPS* Escherichia coli *serotype 055:B5, NO_2_ reductase, o-phenylenediamine (OPD), RPMI-1640 medium, sodium nitrate, and sulphanilamide (FAD) were purchased from Sigma Aldrich Chemical Co. (Toluca, Mexico). Foetal calf serum (FCS) was obtained from Gibco Life Technologies Corporation (Grand Island, NY, USA). L-929 cells were obtained from ATCC (Manassas, VA, USA). The kits with monoclonal antibodies used in the ELISA assay were anti-mouse capture and biotin-labelled detection, and the respective interleukin recombinants were purchased from DB Biosciences Pharmingen (CA, USA). All other reagents were of analytical grade.

## 3. Methods

### 3.1. Plant Material, Extraction, and Identification

*Bougainvillea xbuttiana* (Variety Orange) bracts were authenticated in the HUMO herbarium from Centro de Investigación en Biodiversidad y Conservación (CIByC), UAEM voucher specimen (23683). The extract of* Bougainvillea xbuttiana* (Variety Orange) (BxbO) was maintained, prepared, and standardized as described in patent MX343163B. The chromatographic GC-MS profile of the BxbO extract was previously described by Arteaga Figueroa et al., 2017 [12].

### 3.2. Animals

Female BALB/c mice weighing 20–25 g were acquired from Bioterio del Instituto Salud Publica (Cuernavaca, Morelos, México). All animals were kept within the standards of animal care, and the research protocol was submitted and accredited by the Committee (CCUAL-FM-UAEM N° 005/2016).

### 3.3. Macrophage Preparation

Female BALB/c mice were euthanized following a CCUAL-FM-UAEM N° 005/2016 approved protocol. Peritoneal cells were harvested by peritoneum lavage with RPMI-1640 medium [10]. Peritoneal fluid was pelleted by centrifugation at 350 r.p.m. for 3 minutes. The cells were resuspended at 1 X 10^6^ cells/mL in RPMI-1640 medium enriched with 10% FCS and cultivated in a microplate for 2 hours at 37°C with 5% CO_2_; the nonadherent cells were eliminated by washing with the medium, and the peritoneal cells were as reported in Table 1. All groups of macrophage cultures remained incubated in the same conditions, and at the indicated times, the supernatants were harvested and stored at - 20°C in advance of the quantification of mediators.

### 3.4. Shock Induction and Treatment

#### 3.4.1. LPS Dose Shock Induction

To determine the suitable LPS challenge concentration, groups of mice were intraperitoneally injected with different doses (7.8–62.4 mg/kg). These animals were then observed for 144 hours, and the number of animal deaths was registered daily.

#### 3.4.2. Treatment

To evaluate the protective effect of the BxbO extract against damage by LPS, animals were separated into distinct groups. The BxbO treatment group had 2 mg/kg administered orally, and LPS was administered intraperitoneally. Five groups of mice received all treatments, and at 0, 2, 4, 8, 12, and 24 hours after the various treatments, blood was recovered from the retroorbital plexus and serum obtained was maintained at -20°C for the assessment of the mediators. The other five groups were used for septic survival analysis, and mortality was recorded every 12 hours for 144 hours.  Table 2 describes the treatments performed and the corresponding doses.

### 3.5. Cytokine Quantification

Samples from serum and peritoneal cell supernatants exposed to LPS and/or treated with or without BxbO extract were used to evaluate IL-1*β*, IFN-*γ*, IL-10, and IL-6 levels. These cytokines were evaluated using ELISA kits DB Biosciences Pharmingen (CA, USA) according to the manufacturer's instructions. The levels of sensitivity for all cytokines were 10 pg/mL. To quantify the levels of TNF-*α*, the L929 cell line was used in accordance with the method reported by Ruff and Gifford, 1980 [13].

### 3.6. NO Quantification

The sera samples from the distinct treatments were used to evaluate nitrite production assayed by using Griess reagent [14]. The results were expressed in *μ*M/mL, and the level of sensitivity was 1 nmol.

### 3.7. Data Analysis

Statistical analysis was performed with three repetitions per analysis, and the results were expressed as the mean and standard deviation. Significant discrepancies between samples were determined by the STATISTICA program version 7.0 (STATSOFT 2004), and the variance analysis was determined by Tukey's test at the level of 5% (n = 5;* p* < 0.05).

## 4. Results

### 4.1. Effect of BxbO on Mediator Secretion in LPS-Treated Macrophages

**Figure 1:**
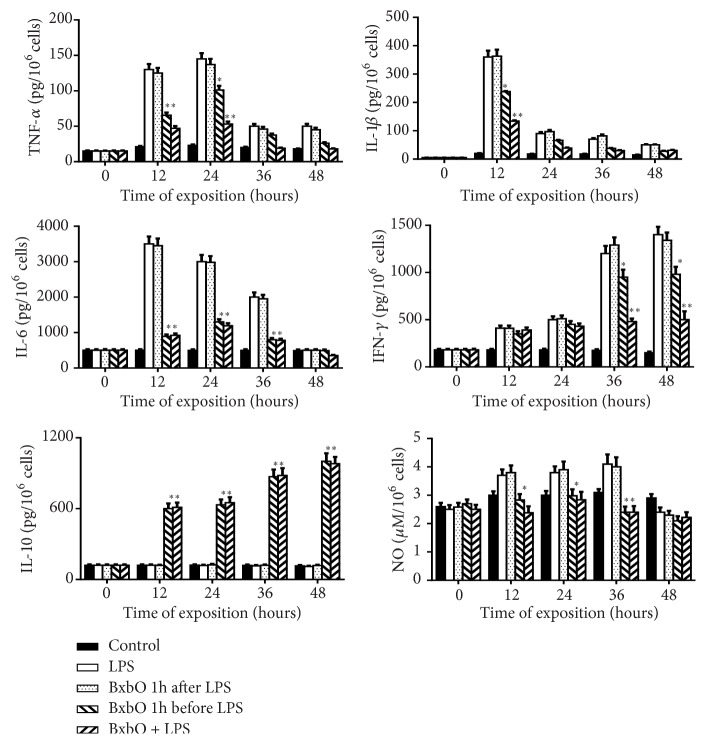
*Effect of BxbO extract on LPS-induced inflammatory cytokine expression in mouse*. Macrophages were treated with LPS (10 *μ*g/100 *μ*L), BxbO 1 hour after LPS challenge, or BxbO 1 before LPS challenge or BxbO plus LPS for 12, 24, 36, and 48 hours. After the indicated time the supernatants were collected, and the levels of mediators were determined as described in Methods. Each bar represents the mean values from 3 different experiments. Statistical significance in comparison with the LPS group is registered, *∗p* < 0.01; *∗∗p* < 0.001.

The effect of the extract on the secretion of inflammatory mediators produced by LPS-treated macrophages was evaluated in cultures treated with the extract 1 hour before, 1 hour after or together with LPS. In previous studies, we have shown that the viability percentages of the macrophages cultures were between 90% for the first 24 hours and 75% from the 48 hours of exposures (data not shown) [10, 12]. In all macrophage supernatants treated with LPS, increased production of proinflammatory mediators was observed. In the supernatants of LPS-treated macrophages, the highest levels of IL-1*β* were observed at 12 hours, for IL-6 and TNF-*α* at 24 hours, for NO at 36 hours, and for IFN-*γ* at 48 hours. In all macrophage cultures treated with the BxbO extract 1 hour after the LPS challenge, the production of mediators was similar to the levels obtained in macrophages treated with LPS (Figure 1). However, in macrophages treated with the BxbO extract 1 hour before or together with LPS, mediator secretion was significantly diminished at all times studied here (*p* < 0.01 and* p* < 0.001, respectively). At the indicated times for each of the mediators, the reductions in the levels of mediator production in macrophages treated with extract 1 hour before or together LPS were, respectively, (a) IL-1*β* (18 hours) 22.2% and 62.8%; (b) IL-6 (24 hours) 56.7% and 60.4%; (c) TNF-*α* (24 hours) 30.4% and 63.5%; (d) NO (36 hours) 41.5% for both treatments; and (e) IFN-*γ* 30.0% and 64.3%. In contrast, in sera from mice treated with LPS 1 hour before or together with the LPS treatment, the levels of IL-10 began to appear at 6 hour and reached the maximum at 24 hours (Figure 1). For the groups of macrophages exposed to the BxbO extract 1 hour before or together with LPS challenge, a significant increase of 5 to 9 times the levels of IL-10 (*p* < 0.001) was observed (Figure 1).

### 4.2. Effect of the BxbO Extract on the Protection of LPS-Challenged Mice

**Figure 2:**
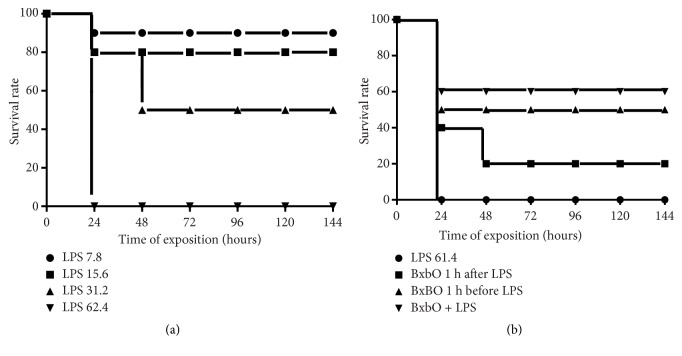
*Effect of BxbO extract on mice protection from LPS-induced septic shock*. (a) Groups of mice were randomly distributed in four different groups which consisted by 5 mice/group and i.p. injected with LPS (with 7.8, 15.6, 31.2, and 62.4 mg/kg) and the survival rates were recorded every 24 hours over a period of 144 hours. (b) Groups of mice were randomly distributed in four different groups and orally treated with 2 mg/kg of BxbO extract at 1 h after or 1 before or together LPS (62.4 mg/kg) and the survival rates were recorded every 24 hours over a period of 144 hours.

With the results obtained from the* in vitro* experiments showing a potent inhibitory effect of the BxbO extract on the mediators secretion in LPS-stimulated macrophages, we decided to extend the study to examine the effect of the extract on endotoxic shock caused by LPS* in vivo*. To establish the best LPS dosage, groups of animals were injected with different doses of LPS and survival was recorded every 12 hours over a period of 144 hours.  Figure 2(a) shows that, in groups of animals injected with 7.8 or 15.6 mg/kg LPS, the survival percentage was 90 and 80%, respectively, and deaths occurred within the first 24 hours after LPS injection. On the other hand, for the groups of mice injected with 31.2 mg/kg, the survival percentage was 20 and 50%, with deaths occurring at 24 and 48 hours, respectively. In the groups of animals treated with 62.4 mg/kg, the survival rate was 0% and all deaths occurred within the first 24 hours (Figure 2(a)).

It was established that the LPS dose of 62.4 mg/kg caused higher mortality, proving the induction of septic shock.  Figure 2(b) shows the effect of the BxbO extract on the protection of mice injected with LPS at 62.4 mg/kg. All female BALB/c mice were treated with the BxbO extract at the dose of 2 mg/kg (via oral administration) 1 hour before, 1 hour after or together with LPS (62.4 mg/kg, intraperitoneal), and survival was monitored daily for a period of 144 hours. Mice groups treated with the BxbO extract 1 hour after challenge with LPS had an observed survival of 40 and 20% at 24 and 48 hours, respectively. Treatment with the BxbO extract 1 hour before the LPS challenge resulted in 50% survival, and all deaths occurred within the first 24 hours. In the groups treated with the BxbO + LPS extract, survival was 60% (Figure 2(b)).

### 4.3. Effect of the BxbO Extract on Mediator Production in Sera from Mice with LPS-Induced Septic Shock

**Figure 3:**
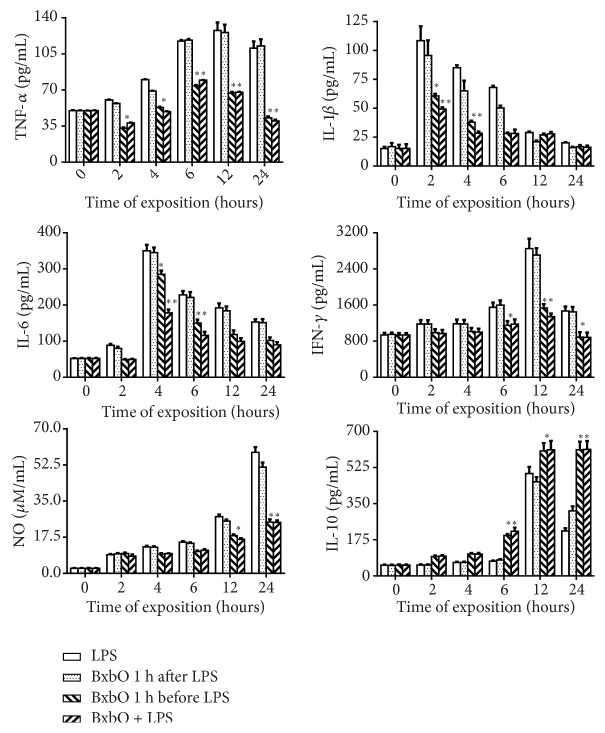
*Effect of BxbO extract on mediator production from LPS-induced septic shock*. Groups of mice were randomly distributed in four different groups which consisted of 5 mice/group and i.p. injected with LPS (31.2 mg/kg) or orally treated with 2 mg/kg of BxbO extract at 1 h after or 1 before or together LPS (31.2 mg/kg) and the mediator production was evaluated at 0, 2, 4, 6, 12, and 24 hours as described in Methods. Each bar represents the standard deviation of 3 different experiments.

The effect of the extract on the secretion of inflammatory mediators produced by the mice treated with LPS was evaluated in sera from mice treated with the extract 1 hour before, 1 hour after or together with LPS. In sera from mice treated with LPS, a significant increment in the secretion of proinflammatory mediators was observed.  Figure 3 shows the kinetics of the production of the mediators. For groups of mice injected with LPS, the highest production of IL-1*β* and IL-6 was observed after 2 and 4 hours of exposure to LPS, respectively. Concerning TNF-*α* levels, the highest levels were detected between 6 and 24 hours subsequently LPS treatment. The highest production of IFN-*γ* and NO was observed at 12 and 24 hours after LPS exposure, respectively. Similar inflammatory mediator production was observed in sera from animals treated with the BxbO extract 1 hour after LPS treatment (Figure 3). In all groups of animals treated with the BxbO extract for 1 hour before or together with LPS challenge, the mediator production in sera decreased (*p* < 0.01 and* p *< 0.001) (Figure 3). Reductions in the levels of mediator production in sera from mice treated with the extract 1 hour before or together with LPS were, respectively, (a) IL-1*β* levels 44.2% and 55%; (b) IL-6 levels 18.6% and 49.2%; (c) TNF-*α* 47.6% and 47.0%; (d) IFN-*γ* 46.2% and 53.0%; and (e) NO 42.3% for both treatments. In contrast, in sera from mice treated with LPS 1 hour before or along with the treatment of LPS, the levels of IL-10 began to appear at 6 hours and reached the maximum at 24 hours (Figure 3). In sera from mice treated with the BxbO extract 1 hour before or together with the LPS challenge, a significant increase of 2.8 times the levels of IL-10 (*p* < 0.001) was observed (Figure 3).

**Figure 4:**
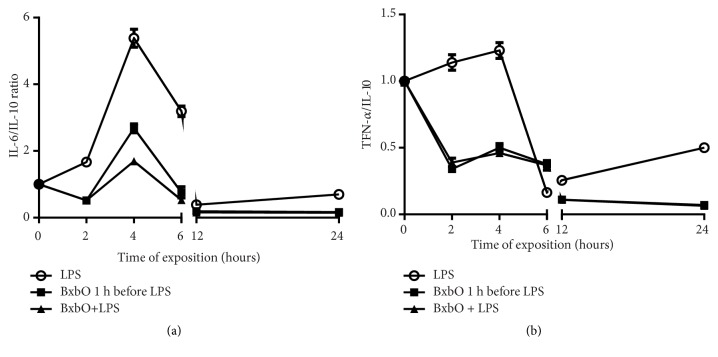
*Effect of BxbO extract on IL-6/IL-10 and TNF-α/IL-10*. (a) The levels of cytokines were evaluated as described in Methods and the IL-6/IL-10 ratios were obtained. (b) TNF-*α*/IL-10 ratios.

Inflammatory biomarkers such as the cytokines IL-6 and TNF-*α* can be used to determine the magnitude of inflammatory responses. In baseline conditions, the IL-6/IL-10 or TNF-*α*/IL-10 ratios should be equal to 1. To demonstrate the protective effect of the BxbO extract, IL-6/IL-10 and TNF-*α*/IL-10 ratios were calculated for the sera of mice. Figures 4(a) and 4(b) show the IL-6/IL-10 and TNF-*α*/IL-10 ratios. The results support the declination of TNF-*α* and IL-6 and the protective effect of IL-10. The IL-6/IL-10 and TNF-*α*/IL-10 ratios observed in the sera of mice treated with the BxbO extract 1 hour before LPS or together with LPS were significantly lower when compared to those obtained in the LPS-treated animals (*p* < 0.001) (Figures 4(a) and 4(b)). These results proved the deterioration of TNF-*α* and IL-6 and the protective effect of IL-10.

## 5. Discussion

We have previously demonstrated that the extract of* B. x buttiana* (Var. Orange) exhibits immunomodulatory activity [10]. We have also demonstrated that* B. x buttiana* extract has the ability to activate murine macrophages in a manner similar to those IL-4 and IL-10 treated macrophages. The effects of this extract on macrophages were low cytotoxicity, increased percentages of vacuolation, hydrogen peroxide production, cell expansion, and phagocytosis [12]. The results obtained in this study demonstrate that the administration of the BxbO extract was able to inhibit the production of inflammatory mediators in both models, macrophages and serum of LPS-treated animals, and was able to guard the host from LPS-induced death. Our results indicated that the inhibition of cytokines by the BxbO extract may be useful as part of a new method for the prevention and treatment of septic shock. When macrophages or animals are injected with LPS, in both macrophages and in serum, they produce various inflammatory mediators, along with TNF-*α*, IL-1*β*, IL-6, IFN-*γ*, and nitric oxide. In this study, the results revealed that the BxbO extract was able to inhibit inflammatory mediators in both models.

In a normal state, the production of these cytokines assists the innate immune response, but in the case of excessive production, it leads to the development of acute-phase endotoxaemia, causing tissue damage, septic shock and death [15, 16]. During the course of an infection, the excessive circulating ranges of TNF-*α*, IL-1*β*, and IL-6 indicate a rapid array of host responses, such as fever and leukocyte chemotaxis. These high concentrations are related to septic shock and multiple organ failure [17–20]. Inhibiting the high levels of TNF-*α* and IL-6 induced by LPS has been a practical tool for the assessment of the anti-inflammatory effects of new drugs [21–23].

The present work showed significant inhibition of IL-1*β*, TNF-*α*, IL-6, and IFN-*γ* levels in both the macrophages and sera of mice treated with the BxbO extract and challenged with LPS. With respect to IL-1, a multifunctional cytokine responsible for host protection, inflammation, and response to injury, LPS-stimulated macrophages produce high IL-1 levels [24–26]. The elevation of IL-1 levels in patients with sepsis is less frequent than the increase in TNF-*α* levels [27, 28]. Another proinflammatory cytokine, IL-6, has a high predictive value for death caused by sepsis [29, 30]. IL-6 is produced by different cell types [31] with levels that are increased from 200 to 2,000 times in patients with septic shock. However, IL-6 can also act as an anti-inflammatory cytokine since it has the ability to induce the release of acute-phase proteins that exert a beneficial effect on sepsis and septic shock. This information confirms that TNF-*α* could be incorporated into an anti-inflammatory strategy and act as a factor of fundamental importance for the treatment of sepsis and the inhibition of these cytokines. In this work, the treatment with the BxbO extract inhibited the production of IL-1*β*, TNF-*α*, and IL-6 in a time-dependent manner. These results suggest that protection in endotoxaemia could be partly attributed to inhibiting the production of these cytokines.

In severe sepsis, changes in protein metabolism with a negative hydrogen balance are associated with an immunodeficient state. Additionally, IL-1 and TNF-*α* are potent inducers of NO inducible synthase gene expression, with a corresponding increase in endothelial production of NO, the primary factor responsible for vascular hypotension that occurs in sepsis [32–34].

In this work, we also found a sizeable increase in IL-10 levels in the two studied models treated with the BxbO extract and challenged with LPS. Among the numerous mediators that intervene in the etiopathology of septic shock, inflammatory cytokines that favour the development of systemic inflammatory processes are highlighted. However, the counterparts to these inflammatory cytokines are also produced in the septic processes; other cytokines, classified as anti-inflammatory cytokines, have the capacity to inhibit the release of proinflammatory cytokines and to counteract or diminish some of the harmful actions and effects that they cause in the development of sepsis. The most important anti-inflammatory cytokines in septic shock are IL-10, IL-4, IL-6, IL-13, granulocyte-macrophage colony stimulating factor IFN-*α*, and transforming growth factor beta. The IL-10 is completely protective in the LPS model of sepsis and the absence of IL-10 leads to more rapid onset of mortality [35, 36]. IL-10 exerts its anti-inflammatory functions on the production of TNF-*α* and produces inhibition of mortality in experimental endotoxaemia [35, 36].

In this study, we also observed that the BxbO extract administered 1 hour before or together with LPS significantly increased IL-10 anti-inflammatory cytokine levels while causing a significant decrease in the levels of proinflammatory cytokines. These results suggest that the BxbO extract has a protective role against sepsis-related inflammation potentially through a shift in cytokines towards anti-inflammatory balance.

In this work, the results obtained* in vivo* seem to be correlated with the* in vitro* results, suggesting that the deactivation of macrophages may be the main mechanism involved in the protective effect of the BxbO extract. In this way, the use of the BxbO extract has the ability to affect the balance between pro- and anti-inflammatory cytokines in sepsis.

The etiopathogenesis of sepsis is not completely understood, although it is related to the elevated production of inflammatory mediators triggering a cascade of activities that contribute to morbidity and mortality [37, 38]. The outcomes of this work suggest that, in addition to the reduction of proinflammatory cytokines, treatment with the BxbO extract was also able to protect animals from endotoxic shock and from the development of clinical signs as a result of LPS, including diarrhoea, piloerection, and lethargy. In the groups of mice administered the BxbO extract 1 hour before or together with LPS, survival was significantly increased.

## 6. Conclusions

Our results demonstrated that the BxbO extract has considerable antiendotoxin activity both* in vitro* and* in vivo*. The extract caused a large decrease in the production of proinflammatory cytokines TNF-*α*, IL-1*β*, IFN-*γ*, IL-6, and NO with a concomitant increase in IL-10. These findings suggest that the BxbO extract modulates inflammatory cytokine production through an IL-10-mediated mechanism.

## Figures and Tables

**Table 1:** Treatment schedule.

Macrophage exposition	Terminology
Control group (saline solution)	C
LPS 10 *μ*g/100 *μ*L	LPS
BxbO 120 *μ*g/100 *μ*L	BxbO
BxbO 120 *μ*g/100 *μ*L 1 hour before LPS 10 *μ*g/100 *μ*L	BxbO 1 hour before LPS
BxbO 120 *μ*g/100 *μ*L 1 hour after LPS 10 *μ*g/100 *μ*L	BxbO 1 hour after LPS
BxbO 120 *μ*g/100 *μ*L + LPS 10 *μ*g/100 *μ*L	BxbO + LPS

**Table 2:** Treatment schedule.

Group (n=5)	Treatment (mg/kg)
1	LPS (31.2)
2	BxbO (2)
3	BxbO (2) 1 before LPS (31.2)
4	BxbO (2) 1 after LPS (31.2)
5	BxbO (2) + LPS (31.2)

## Data Availability

The data used to support the findings of this study are available from the corresponding author upon request.

## Abbreviations

LPS: Lipopolysaccharide
TNF-*α*: Tumour necrosis factor
IL-1*β*: Interleukin-1 beta
IL-6: Interleukin-6
IFN-*γ*: Interferon-gamma
NO: Nitric oxide.
